# Insulin Pump-Induced Lipoatrophy: Proposed Diagnostic and Management Algorithm

**DOI:** 10.1210/jcemcr/luaf168

**Published:** 2025-07-31

**Authors:** Harsan Kanagaretnam, Kajanan Parameshwaran, Avinash Suryawanshi

**Affiliations:** Endocrinology Department, Concord Repatriation General Hospital, Concord, New South Wales 2139, Australia; The University of Sydney Concord Clinical School, Sydney, New South Wales 2139, Australia; Endocrinology Department, Concord Repatriation General Hospital, Concord, New South Wales 2139, Australia; Endocrinology Department, Concord Repatriation General Hospital, Concord, New South Wales 2139, Australia

**Keywords:** insulin pump-induced lipoatrophy, lipoatrophy, insulin pump complications, subcutaneous fat loss, type 1 diabetes

## Abstract

Lipoatrophy, the localized loss of subcutaneous fat, is a rare complication of insulin therapy that persists despite advancements in insulin formulations and delivery systems. This case report describes a 58-year-old woman with type 1 diabetes mellitus who developed considerable abdominal wall lipoatrophy 4 months after initiating insulin pump therapy. Management included changing the infusion cannula type, rotating insertion sites, and switching insulin analogues, resulting in stabilization and complete improvement of lipoatrophy over 16 months. A diagnostic and management algorithm is proposed, incorporating site rotation, cannula and insulin type modifications, and potential adjunctive therapies including topical sodium cromoglycate and corticosteroids.

## Introduction

Lipoatrophy, a localized loss of subcutaneous fat, is a recognized complication of insulin therapy, affecting approximately 1% of patients with type 1 diabetes mellitus (T1DM) [1]. This phenomenon, which can affect insulin absorption and glycemic control, has become much less common with the switch from nonhuman insulins to recombinant human insulin and insulin analogues [2]. Nonetheless, cases continue to emerge, including in patients using insulin pumps [1-8]. We propose a systematic approach to guide clinicians in addressing insulin-induced lipoatrophy effectively based on published case reports in the literature.

## Case Presentation

Two years before the current evaluation, a 58-year-old woman was diagnosed with T1DM when she presented with diabetic ketoacidosis. At diagnosis, she had detectable antiglutamic acid decarboxylase antibodies at 102.5 U/mL (normal range, 0-0.9 U/mL), a low connecting-peptide (C-peptide) level of 136 pmol/L (200-1200 pmol/L), and elevated glycated hemoglobin A_1c_ (HbA_1c_) of 11.2% (aim < 7%). Her past medical history was significant for Hashimoto thyroiditis, osteoporosis, cholecystectomy, hysterectomy with ovarian conservation, and treated left breast cancer. She did not have any other autoimmune disease. Her medications included zoledronic acid 5 mg yearly, cholecalciferol 25 mcg daily, iron polymaltose 370 mg daily, and levothyroxine 50 and 100 mcg alternate-day doses.

She was initially managed with a subcutaneous basal-bolus insulin regimen using insulin glargine and insulin aspart. Ten months after diagnosis, she transitioned to a T-slim X2 insulin pump delivering insulin aspart using the Control-IQ closed-loop system with a Dexcom continuous glucose monitor. Her HbA_1c_ improved from 8.9% to 6.7% after 3 months of transitioning to insulin pump therapy.

**Figure 1.**
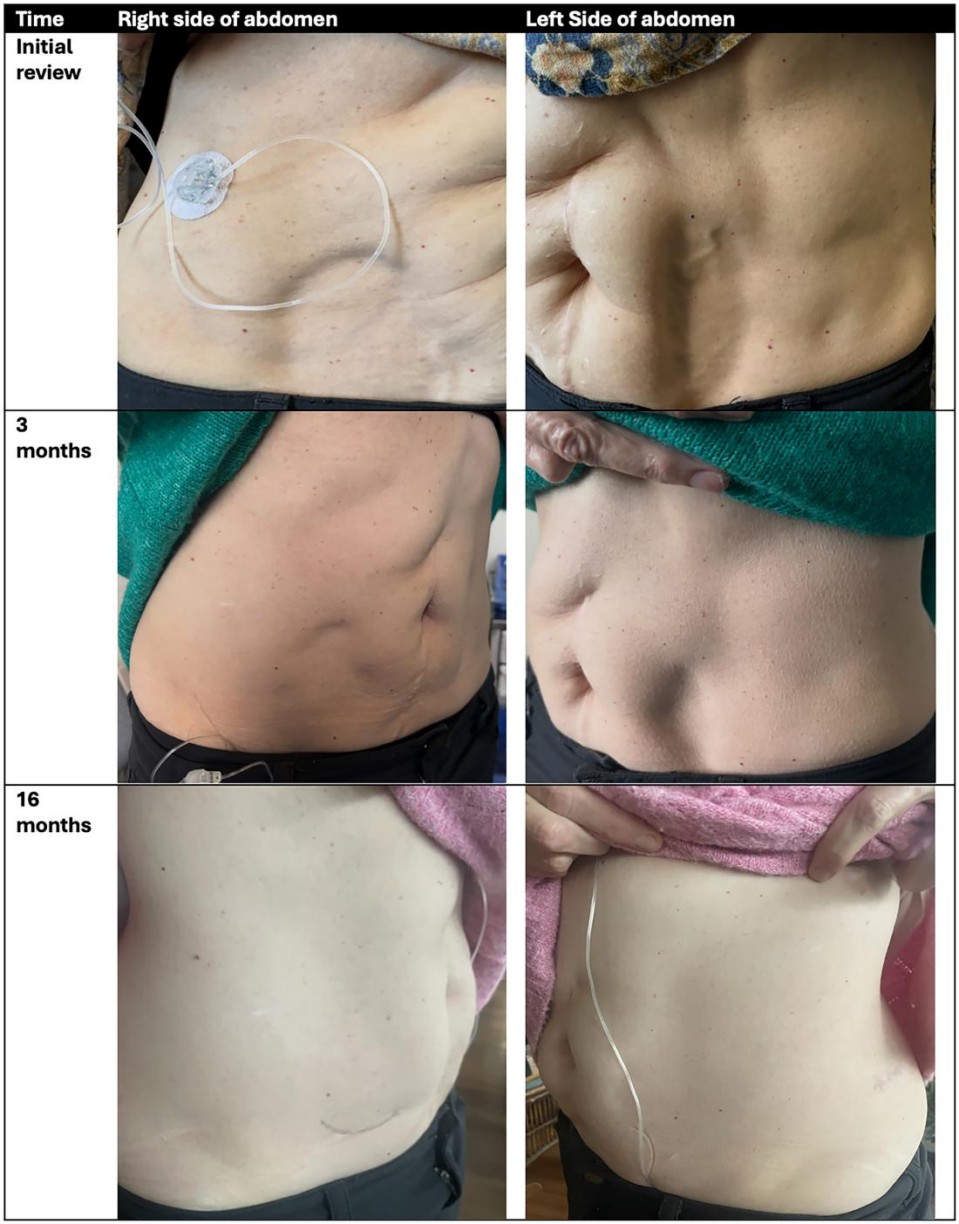
Photos of lipoatrophy. Initial photos showing areas of lipoatrophy in the right lower quadrant, left lower quadrant, and left lateral abdomen, where the patient had inserted the insulin pump cannula. Photos of lipoatrophy 3 months later (after changing from a steel to a Teflon cannula and changing the insulin within the pump from insulin aspart to insulin lispro) demonstrate marked improvement in the area of lipoatrophy. Photos at 16 months show complete resolution.

She changed her insulin pump set every 2 days and rotated the injection site as directed. After 4 months of insulin pump therapy, she noticed increased indentations at the canula insertion sites of her anterior abdomen and posterior lumbar region, reflecting the development of considerable abdominal wall lipoatrophy at these sites (Fig. 1).

## Diagnostic Assessment

**Figure 2.**
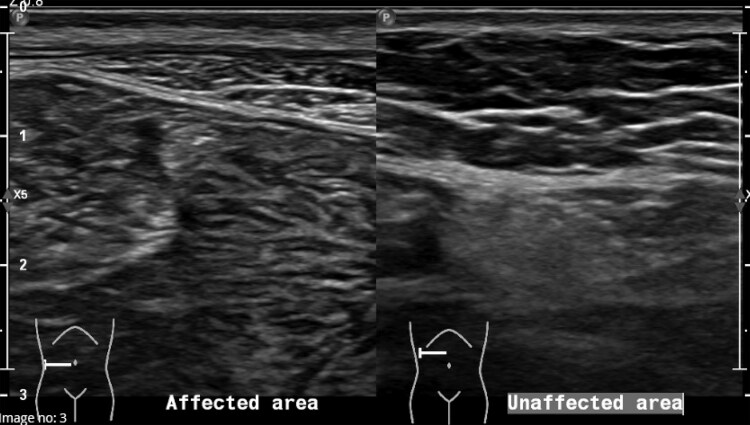
Ultrasound images. Ultrasound of the abdominal wall in the right lower quadrant shows that the affected lipoatrophic area has 3 mm of subcutaneous fat, whereas the unaffected adjacent region has 11 mm of subcutaneous fat.

An abdominal ultrasound confirmed the areas of lipoatrophy (Fig. 2). She was evaluated in a dermatology clinic, and incisional biopsies of the most recent areas of lipoatrophy were performed. Histopathology revealed lipoatrophy involving most of the biopsy, with lipocytes reduced in size and rounded in appearance, without inflammation. There was transition to normal adipose tissue at the nonlesional end.

## Treatment

Two months after the initial development of lipoatrophy, the patient changed the infusion cannula from steel to an AutoSoft Teflon cannula, with substantial improvement in the region of lipoatrophy. However, given the persistence of some abdominal wall lipoatrophy, she switched her insulin infusion site to her thighs. The insulin in the pump was also changed to insulin lispro approximately 2 weeks after changing the insulin infusion site to her thighs.

## Outcome and Follow-up

Five months after the initial development of lipoatrophy, she had developed no new areas of lipoatrophy and the existing areas of abdominal wall atrophy improved over time. The infusion site was kept on the thighs to allow the abdominal areas to heal. Glycemic control remained excellent with an HbA_1c_ of 6.8% without hypoglycemia. Nine months after the initial development of lipoatrophy, the areas of lipoatrophy had continued to improve and she returned to using her lower back as the insulin infusion site. Sixteen months after the initial development of lipoatrophy, all areas of lipoatrophy had completely resolved. She continues to use insulin lispro in her insulin pump, and the lower back remains the site of insulin infusion.

## Discussion

The exact pathophysiology of insulin-induced lipoatrophy is not fully understood, although an immune complex–mediated process has long been postulated. Historically, insulin-induced lipoatrophy was a common occurrence with the use of animal-derived insulins, such as bovine and porcine insulin, which were believed to trigger the production of antibodies that would form complexes with the injected insulin [9]. Indeed, skin biopsies performed on patients with lipoatrophy from bovine or porcine insulin have shown abnormal deposition of immunological components (immunoglobulin M [IgM] and C3), suggesting local immune complex formation, that is, a type III hypersensitivity reaction [9]. The introduction of recombinant human insulin in the 1980s, and the subsequent development of human insulin analogues, significantly reduced the incidence of lipoatrophy due to their lower antigenicity [9]. Nonetheless, there continue to be infrequent cases of lipoatrophy associated with recombinant human insulin or human insulin analogues (Table 1), regardless of delivery via continuous subcutaneous insulin infusion (CSII) or multiple daily injections (MDIs).

**Table 1.** Case reports of lipoatrophy secondary to subcutaneous insulin*^a^*

Study	Patient age, sex	CSII*^b^* or MDI*^c^*	Insulin type	LA details	Treatment	Outcome
Xatzipsalti et al [1]	7 M, 6 F. Age range 2-24 y, median 9.6 y	CSII – 9 MDI – 4	This study evaluated interventions for LA in 13 patients with T1DM, including 10 on CSII and 3 on MDIs SCG cream used in 10 patients, with 7 showing improvement (2 complete, 5 partial) over 3 mo to 2 y Among these, 3 combined SCG with a switch to glulisine insulin, resulting in 2 complete and 1 partial resolution, though laser treatment contributed in 1 case Changing insulin type alone led to partial or complete improvement in 5 patients, including 2 who self-improved over time. One patient receiving a single percutaneous betamethasone injection showed improvement on treated side but not on untreated side. Laser therapy combined with SCG was highly effective in 2 patients, yielding substantial resolution, while 5 patients experienced self-improvement over 1-7 y			
Griffin et al [2]	10 y, F	CSII	Insulin lispro (Humalog)	LA of anterior abdominal wall starting 12 mo after initiation of CSII	Switch to buffered human regular insulin (Velosulin)	No further progression; persistence of existing areas at publication
Griffin et al [2]	51 y, F	CSII	Insulin lispro (Humalog)	LA at injection sites (abdominal wall, lateral thigh, buttock) at ∼12 mo following change from regular human insulin to insulin lispro	Switch to buffered human regular insulin (Velosulin)	No further progression of LA at time of case report publication
Ampudia-Blasco et al [3]	29 y, F	CSII	Human regular insulin (Actrapid), Insulin lispro	LA of anterior abdominal wall, 7 mo after initiation of CSII with human regular insulin. New area of LA of right buttock, 11 mo after change of CSII to insulin lispro but it was of lesser extension	Frequent change of catheter site every 3 d; patient prompted to avoid abdominal area for catheter insertion Treatment also changed from buffered regular human insulin to lispro insulin 10 mo later due to deterioration of metabolic control	No further progression of LA and persistence of existing areas at time of case report publication
Boland et al [4]	18 y, F	CSII	Insulin aspart (Novorapid)	Lipoatrophy of right abdominal wall noticed after 1 y of CSII	Avoided area of LA and rotated to other sites	Original area of LA did not improve and did not progress. No further areas of LA at other sites
Chantelau, Praetor et al [5] and Chantelau, Prätor, Prätor [7]	53 y, F	CSII	Humalog, Actrapid	LA right side of abdominal wall, and left-sided abdominal wall after switching cannula site	Change to human insulin Actrapid showed no benefit. Prednisone 10 mg daily slowly weaned to 2.5 mg resulted in no new LA and previous areas of LA improved. Patient switched back to humalog while on prednisone and LA improved. There was relapse 4 wk after cessation of prednisone. Recommenced prednisone 10 mg treatment for 2 mo and prednisone tapered off with resolution of LA. Two years later she had another relapse following shingles. Prednisone 10 mg/d was given for 2.5 mo, tapered to 5 mg/d for 1.5 mo, and then stopped, achieving partial improvement. Prednisone 10 mg/d was restarted for 5 mo to fully resolve LA, which remained in remission 1 y later	
Lopez et al [6]	3 F, 2 M patients	CSII – 3 MDI – 2	Insulin aspart (n = 4), lispro (n = 4), NPH (n = 3), glargine (n = 1), and regular (n = 1).	All 5 patients received topical 4% sodium cromolyn, applied twice daily, for an average of 12 wk (range, 4-20 wk). None of the patients developed new LA lesions at new insulin injection sites after starting treatment with cromolyn. Four of 5 patients showed significant improvement in existing LA lesions (1 had complete resolution). There was no improvement in 1 patient		
Milan et al [8]	N/A	CSII	Various insulin analogues	LA induced by subcutaneous insulin infusion	Ultrastructural analysis and gene expression profiling	Analysis of LA mechanisms, suggesting immune response
Babiker, Datta [10]	4 y, F	MDI	Biphasic insulin aspart (NovoMix 30)	LA at injection sites in upper arm and leg	Changing injection site	Resolution of LA over 1-2 y
Babiker, Datta [10]	5 y, F	MDI	Biphasic insulin aspart (NovoMix 30)	LA at injection sites, with new region of LA developing on changing injection site	Switch to biphasic insulin lispro (Humalog Mix 25)	Resolution of LA over 1-2 y
Arranz et al [11]	45 y, F	MDI	Insulin lispro (in addition to longstanding use of NPH insulin)	LA on right thigh, 23 mo after initiating insulin lispro therapy. Subsequent development of similar lesion on contralateral thigh after 6 mo of switching to this site	Switch from insulin lispro to insulin aspart	No further progression of LA and persistence of existing areas at time of case report publication
Phua et al [12]	N/A	MDI	insulin aspart (n = 4), lispro (n = 5), regular human insulin (n = 1), NPH insulin (n = 1), glargine (n = 2), and detemir (n = 1), with 3 patients reporting use of >1 insulin preparation	10 patients with LA used topical cromolyn sodium. 6 had complete resolution and 4 had partial resolution There were 14 patients in this series who did not use topical cromolyn sodium. − 10 patients who changed insulin preparation: 2 had complete resolution, 6 had partial resolution, 2 had no change − 4 patients switched to continuous subcutaneous insulin infusion: 2 had complete resolution and 2 had no change − 2 patients used glucocorticoids (unspecified regarding type and dose of glucocorticoid): 1 had complete resolution and 1 had no change − 1 patient had spontaneous complete resolution Noncromolyn users exceeded 14 cases, as patients may have used >1 treatment option		
Ramos, Farias [13]	4 y, F	MDI	NPH insulin	LA areas affecting multiple injection areas, most notably on arms	Injection of betamethasone (0.075 mg), mixed with usual insulin dose into affected area	Total remission of LA within 6 mo of steroid treatment
Kumar et al [14]	Ages: 13, 23, 51, 21, 35, 54, 10, 22, 30. All F	MDI	Not specified	All patients had LA with bilateral involvement of thighs. Single-blinded study with 1 thigh injected with insulin NPH in 1 thigh (control) and insulin mixed with dexamethasone in other thigh (treatment). Six cases had significant improvement in filling of LA areas of treatment side. At 6 mo, 3 cases had not shown any benefit		
Mu, Goldman [15]	47 y, F	MDI (T2DM)	Human regular insulin (Humulin N)	LA of upper arms, and subsequently thighs, ∼12 mo after commencing insulin therapy	Cessation of insulin therapy	No further progression of LA and persistence of existing areas; patient subsequently lost to follow-up

^*a*^This table shows results of these studies summarized with key results only. For further details regarding these studies, please refer to referencing as indicated.

Abbreviations: CSII, continuous subcutaneous insulin infusion (eg, from insulin pumps); F, female; LA, lipoatrophy; M, male; MDI, multiple daily injections of insulin; N/A, not available; NPH, neutral protamine Hagedorn; SCG, sodium cromoglycate; T1DM, type 1 diabetes mellitus; T2DM, type 2 diabetes mellitus.

In 2008, Lopez et al described histopathology from skin biopsies of 4 patients with recombinant human insulin–induced lipoatrophy [6]. All 4 biopsies demonstrated elevated populations of degranulated mast cells, as well as prominent eosinophils in 3 out of 4 cases, while there was no deposition of IgM, IgA, IgG, C3, or fibrin. These results were indicative of an allergy-mediated immune response, rather than one driven by immune-complex deposition. Unfortunately, there have not been any other published studies reporting histopathologic analysis of lipoatrophy to corroborate these findings and, in this case report, dedicated staining for immunoglobulin or complement deposition was not performed.

Other postulated mechanisms for lipoatrophy with recombinant insulin or insulin analogues include mechanical trauma from repeated injections and cryotrauma. This is based on the associations between repeated use of the same insulin injection site or same device and increased risk of lipoatrophy [16].

**Figure 3.**
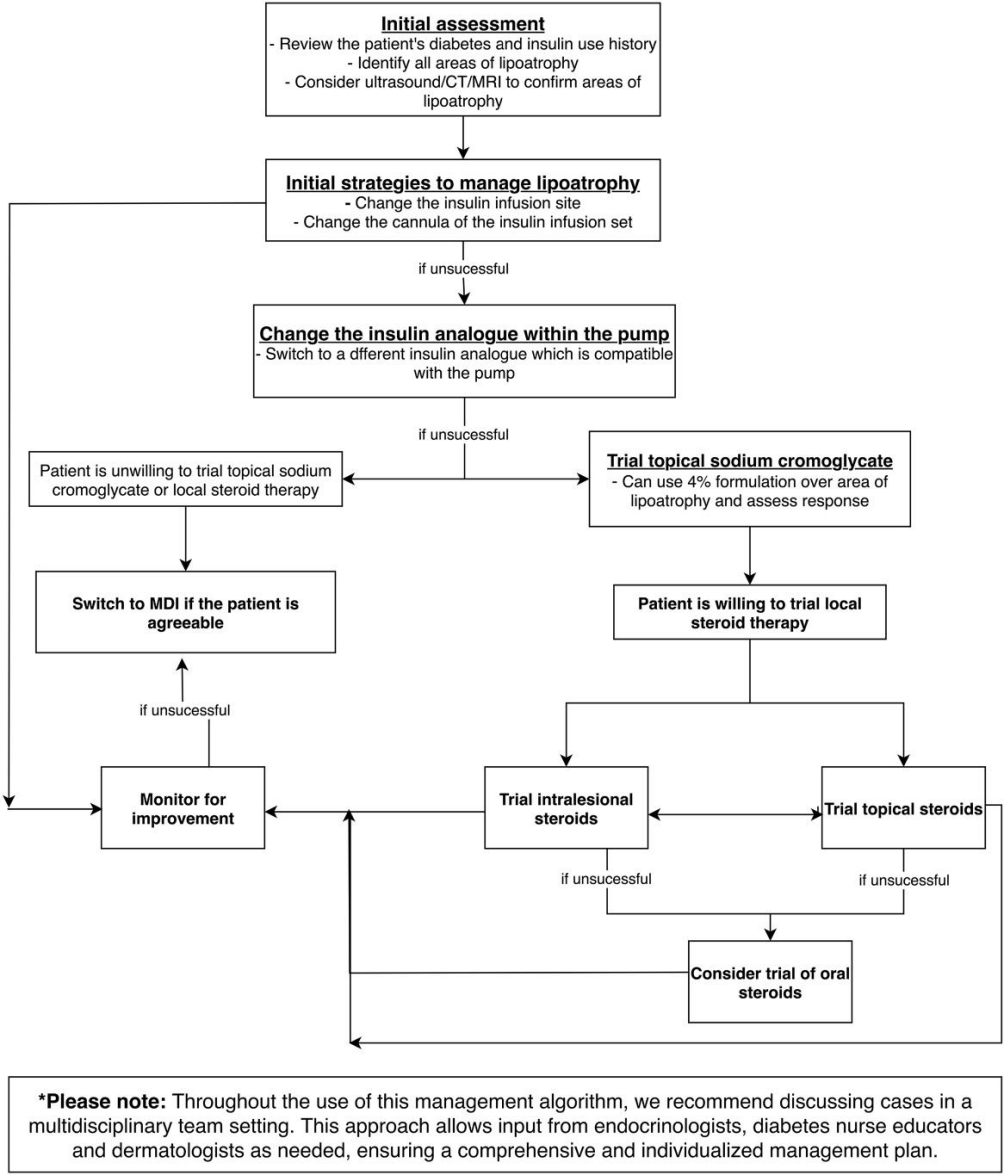
Diagnostic and management algorithm. Flowchart demonstrating a diagnostic and management algorithm for insulin pump-induced lipoatrophy.

A literature review identified 16 case reports, case series, or research articles reporting insulin-related lipoatrophy (see Table 1). Based on our case presentation and review of the literature, the following diagnostic and management algorithm is proposed for patients with insulin pump–induced lipoatrophy (Fig. 3). We recommend discussing cases in a multidisciplinary team setting whenever possible, given the rarity of this condition. Input should be sought from endocrinologists, diabetes nurse educators, and dermatologists to develop a comprehensive and individualized management plan.

Initial assessment should include a review of the diabetes and insulin history. Note the onset of lipoatrophy and prior management attempts with their outcomes. Identify and document the areas of lipoatrophy. With consent, photograph the affected areas for ongoing comparison in medical records. Clinical examination is typically sufficient for identifying lipoatrophy, but radiological confirmation can be sought with ultrasonography, computed tomography (CT), or magnetic resonance imaging (MRI). Lipoatrophy appears on ultrasound as a focal area of decreased subcutaneous fat thickness and increased heterogeneous echogenicity compared to surrounding tissue [8]. Ultrasonography compares well with CT and MRI for the purposes of evaluating subcutaneous lipoatrophy, while being more accessible and safer than the latter modalities [17].

Initial strategies to manage lipoatrophy should include changing the insulin infusion site every 2 to 3 days [1, 4]. If this is unsuccessful, consider changing the cannula of the insulin infusion set. There are 2 types of cannulas used with insulin infusion sets: Teflon and steel cannulas. Teflon cannulas are flexible and generally more comfortable than steel cannulas and are less likely to cause tissue damage or irritation. While there were no case reports describing the use of this strategy, our patient reported substantial improvement after switching from a steel to a Teflon cannula.

Using a different insulin analogue within the pump should also be considered. Some case reports have reported improvement or stabilization in areas of lipoatrophy when the insulin type delivered by CSII [2, 3] or MDI [10, 11] was changed. However, a single-center case series presented by Xatzipsalti et al [1] highlights that this strategy is only variably successful. They reported on 13 pediatric patients who developed insulin-induced lipoatrophy, with all but 1 patient trialing a change in insulin type. Two patients using CSII demonstrated improvement in lipoatrophy after switching from aspart to glulisine, and one patient had improvement after changing from lispro to glulisine. In contrast, many patients using CSII had no improvement after switching insulins: Two patients switched from lispro to aspart; 2 switched from aspart to lispro; and 1 switched from aspart to glulisine and returned to aspart without improvement. In a case series described by Phua et al [12], 10 patients who had MDI insulin-induced lipoatrophy switched the insulin preparation. This resulted in complete resolution for 2 patients, partial resolution for 6 patients, and no change for 2 patients. In this same case series, 4 patients using MDI switched to CSII. Of these patients, 2 had complete resolution of lipoatrophy while the other 2 had no change (whether the type of insulin used was changed was not mentioned). It should be noted that only certain types of insulin are compatible with each insulin pump. In our case report, pump insulin was changed from aspart to lispro. Subsequently, the patient had no new areas of lipoatrophy and existing areas of lipoatrophy completely healed.

Another management option is a trial of sodium cromoglycate (SCG). SCG is an anti-inflammatory medication that inhibits the release of inflammatory mediators from mast cells. In the case series described by Xatzipsalti et al [1], 10 patients used topical SCG cream (4% formulation in petrolatum solvent); 9 of these patients also changed insulin type. Seven patients showed improvement within a period ranging from 3 months to 2 years (median 8 months). Of note, there was a clear, beneficial effect of SCG in 4 patients who did not respond to change of insulin type. In the case series by Phua et al [12], 6 of 10 patients with lipoatrophy who used topical SCG had complete resolution, while 4 had partial resolution. In the case series described by Lopez et al [6], topical SCG resulted in significant improvement in lipoatrophy for 4 of 5 patients. Topical SCG provides a steroid-sparing therapeutic option for management of insulin-induced lipoatrophy.

Corticosteroids can be considered for resistant lipoatrophy. Topical steroids, intralesional subcutaneous corticosteroid injections, and oral prednisone have all been trialed in the management of insulin-induced lipoatrophy. The beneficial effect of intralesional subcutaneous steroids is described in one case report and one case series [13, 14]. Kumar et al [14] reported on 9 patients with MDI-associated bilateral thigh lipoatrophy who received insulin neutral protamine Hagedorn (NPH) mixed with dexamethasone in 1 affected thigh. Six patients had significant improvement in their lesions. The effective use of oral prednisone was described in 1 patient using CSII across 2 case reports [5, 7]. This patient had 2 episodes of lipoatrophy with each relapse being treated with a tapering course of prednisolone to good effect. We recommend trialing topical or intralesional subcutaneous corticosteroids prior to oral steroids due to a reduced risk of systemic side effects and likely similar efficacy.

The role of biopsy to guide management of lipoatrophy is unclear. The presence of inflammatory cells on histopathology may justify a trial of local or systemic steroid therapy. Nonetheless, there is a lack of evidence to support a strong recommendation for biopsy.

Finally, a switch from insulin pump to MDI can be considered at any stage in the management process. However in T1DM, MDI is associated with less time in range, higher HbA_1c_, and increased risk of hypoglycemia when compared with automated closed-loop insulin [18].

This case highlights the complexity of managing insulin-induced lipoatrophy and underscores the need for a systematic approach. Further research is essential to validate these strategies, standardize diagnostic and therapeutic protocols, and explore the role of adjunctive treatments, including SCG and corticosteroids.

## Learning Points

- Lipoatrophy is a complication of insulin therapy that may be immune-complex mediated or an allergy-mediated immune response.
- Initial strategies for managing insulin pump-induced lipoatrophy include changing the infusion site, cannula type, and insulin analogue within the pump.
- If these simple strategies are unsuccessful, adjunctive therapies include topical SCG, topical steroids, intralesional subcutaneous steroids, or oral steroids.

## Contributors

All authors made individual contributions to authorship. H.K. and A.S. were involved in the diagnosis and management of the patient. H.K. was involved in the conceptualization, literature review, write-up, editing, project, and submission of the journal article. K.P. was involved with the literature review, write-up, and editing. A.S. was involved with conceptualization, editing, and supervision. All authors reviewed and approved the final draft.

## Data Availability

Original data generated and analyzed during this study are included in this published article.

## Abbreviations

CSII: continuous subcutaneous insulin infusion (eg, from insulin pumps)
CT: computed tomography
HbA_1c_: glycated hemoglobin A_1c_
IgM: immunoglobulin M
MDI: multiple daily injections of insulin
MRI: magnetic resonance imaging
NPH: neutral protamine Hagedorn
SCG: sodium cromoglycate
T1DM: type 1 diabetes mellitus
